# Multifaceted Role of *PheDof12-1* in the Regulation of Flowering Time and Abiotic Stress Responses in Moso Bamboo (*Phyllostachys edulis*)

**DOI:** 10.3390/ijms20020424

**Published:** 2019-01-19

**Authors:** Jun Liu, Zhanchao Cheng, Lihua Xie, Xiangyu Li, Jian Gao

**Affiliations:** International Center for Bamboo and Rattan, Key Laboratory of Bamboo and Rattan Science and Technology, State Forestry Administration, Beijing 100102, China; liujun_0325@163.com (J.L.); chengzhan_chao@126.com (Z.C.); xielihua0227@163.com (L.X.); leerduo727@163.com (X.L.)

**Keywords:** *Phyllostachys edulis*, Dof transcription factor, flowering time, abiotic stress, gene expression

## Abstract

DNA binding with one finger (Dof) proteins, forming an important transcriptional factor family, are involved in gene transcriptional regulation, development, stress responses, and flowering responses in annual plants. However, knowledge of Dofs in perennial and erratically flowering moso bamboo is limited. In view of this, a Dof gene, *PheDof12-1*, was isolated from moso bamboo. *PheDof12-1* is located in the nucleus and has the highest expression in palea and the lowest in bract. Moreover, *PheDof12-1* expression is high in flowering leaves, then declines during flower development. The transcription level of *PheDof12-1* is highly induced by cold, drought, salt, and gibberellin A3 (GA_3_) stresses. The functional characteristics of *PheDof* are researched for the first time in Arabidopsis, and the results show that transgenic Arabidopsis overexpressing *PheDof12-1* shows early flowering under long-day (LD) conditions but there is no effect on flowering time under short-day (SD) conditions; the transcription levels of *FT*, *SOC1*, and *AGL24* are upregulated; and *FLC* and *SVP* are downregulated. *PheDof12-1* exhibits a strong diurnal rhythm, inhibited by light treatment and induced in dark. Yeast one-hybrid (Y1H) assay shows that *PheDof12-1* can bind to the promoter sequence of *PheCOL4*. Taken together, these results indicate that *PheDof12-1* might be involved in abiotic stress and flowering time, which makes it an important candidate gene for studying the molecular regulation mechanisms of moso bamboo flowering.

## 1. Introduction

DNA binding with one finger (Dof) transcription factors (TFs) are a family of plant-specific transcription factors. The proteins generally contain 50–52 highly conserved amino acids, including a C_2_C_2_-type zinc-finger motif at the N-terminal end [1]. Dof transcription factors have been shown to be widely distributed in the plant kingdom. The cDNA sequence of Dof was first obtained from *Zea mays* [2]. Since then, many Dofs have been cloned from various plant species [3,4,5]. In previous studies, it is suggested that Dof proteins are involved in the regulation of a variety of biological processes, including seed germination, floral organ abscission, hormone signaling, and cell cycles. In *Arabidopsis*, *DAG1* and *DAG2* can promote seed germination [6,7], *DOF6* acts as a negative regulator of seed germination and interacts with TCP14 [8], and *AtDOF4.7* participates in the transcriptional regulation of floral organ abscission via an effect on cell wall hydrolase gene expression [9]. In addition, some Dof genes (*AtDof2.4*, *AtDof5.8*, and *AtDof5.6*/*HCA2*) are expressed in the early development of vascular cells [10]. In rice, *OsDof3* is involved in gibberellin-regulated expression [11]. Moreover, Dof TFs such as maize *Dof1* and *Dof2* are also involved in the control of carbon and nitrogen metabolism through the regulation of phosphoenolpyruvate carboxykinase (PECPK), glutamine synthase (GS), and glutamate synthase (GLU) [7,12,13,14,15,16].

Genetic and molecular studies have suggested that Dof transcription factors participate in different stresses, light responsiveness, and flowering regulation. In *Brachypodium distachyon*, *BdCBF1*, *BdCBF2*, and *BdCBF3* contribute to cold, drought, and salt stresses by regulating downstream targets such as DEHYDRIN5.1 (*Dhn5.1*) and *COR* genes [17]. Overexpressing *SlCDF3* shows increased transgenic Arabidopsis drought and salt tolerance [18]. In Chinese cabbage, most *BraDof* genes are induced by cold, heat, high salinity, and drought stresses [19]. Moreover, Dof proteins are involved in photoperiod flowering. In Arabidopsis, cycling Dof factor-1 (CDF1) binds to the *COSTANS* (*CO*) and *FLOWERING LOCUS T* (*FT*) promoter regions to block transactivation of these two flowering genes, whereas this inhibition could be released based on the GIGANTEA-FLAVIN-BINDING, KELCH REPEAT, F-BOX1(GI-FKF1) complex-mediated degradation of CDF1 under long-day (LD) conditions [20]. In addition, *CDF2*, *CDF3*, and *CDF5* repress flowering of Arabidopsis by decreasing the mRNA level of *CO* [21]. In rice, overexpressing *OsDof12* promotes early flowering under LD conditions by upregulating the expression of *Hd3a* and *OsMADS14* [22]. Although a large number of Dofs have been extensively studied in annual plants [23,24], the knowledge of Dofs in moso bamboo is limited.

Moso bamboo (*Phyllostachys edulis*) is a perennial plant characterized by a long vegetative stage that flowers synchronously followed by widespread death [25]. In this case, studying the mechanism of moso bamboo flowering time is very important and challenging, and it is quite difficult to determine the key regulatory gene. Moreover, the growth of moso bamboo in the wild is severely threatened by various environmental conditions such as drought, salinity and cold, which severely limit the growth and distribution of moso bamboo and affect the yield and quality of winter shoots, as well as new bamboo yield in the following year and the yield of wood harvesting of the subsequent years [26,27,28]. In addition, recent research on Dofs is mainly in annual plants, and is limited in perennials. Therefore, researching the role of Dofs in moso bamboo is necessary, especially in terms of abiotic stress and flowering time. In this study, a Dof gene (*PheDof12-1*) is isolated from moso bamboo, induced by cold, drought, salt, and gibberellin (GA_3_) stresses. The functional characteristics of *PheDof12-1* are researched for the first time by ectopic expression in Arabidopsis, and transgenic Arabidopsis overexpressing homozygous *PheDof12-1* show early flowering under long-day (LD) conditions, binding to the promoter sequence of PheCOL4 with a strongly diurnal pattern. These results provide new insights into the functions of the Dof transcription factor in the regulation of photoperiod flowering time and abiotic stress in moso bamboo.

## 2. Results

### 2.1. Isolation and Analysis of PheDof12-1

**Figure 1:**
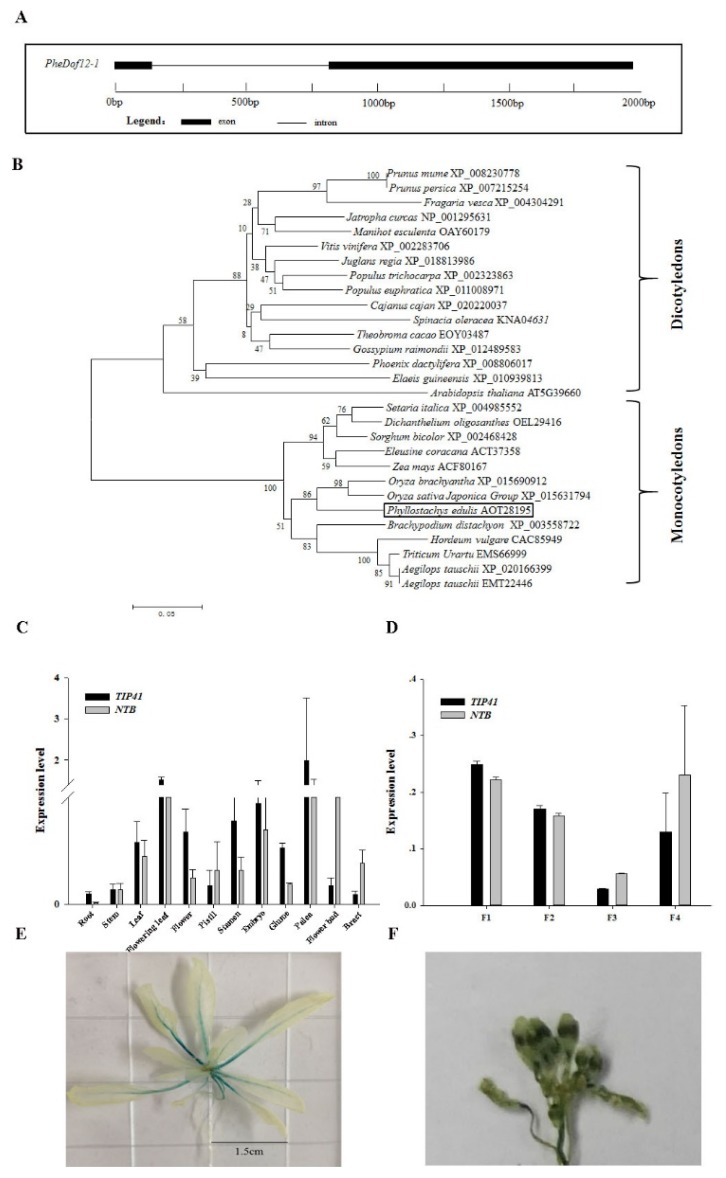
Characterization and preliminary expression analysis of *PheDof12-1*. (**A**) Gene structure of *PheDof12-1*. (**B**) Phylogenetic analysis of PheDof12-1 with other DNA binding with one finger (Dof) proteins. (**C**) qRT-PCR analysis of *PheDof12-1* in different tissues of moso bamboo. (**D**) Expression profile of *PheDof12-1* in different flower developmental stages: F1: floral bud formation stage; F2: inflorescence growing stage; F3: blooming stage; F4: flowers are withered. (**E**) Glucuronidase (GUS) staining of *Pro_PheDof12-1_*-*GUS* in transgenic Arabidopsis seedling. (**F**) GUS staining of *Pro_PheDof12-1_*-*GUS* plants showing PheDof12-1 localization in flower and pollen.

Based on the moso bamboo genome database, *PheDof12-1* was isolated from moso bamboo. The full-length CDS of *PheDof12-1* is 1299 bp, encoding 432-amino acids, with predicted molecular weight (MW) and isoelectric point (pI) of 46.37 kDa and 8.32, respectively. Structure analysis showed that PheDof12-1 contains one intron and two exons (Figure 1A). The deduced proteins contain the conserved zf-Dof domain. Furthermore, phylogenetic analysis of PheDof12-1 and homologous proteins from other plants shows that PheDof12-1 and other Dofs from monocotyledons belong to the same clade (Figure 1B). The amino acid sequence of PheDof12-1 shows 83% and 84% identity with *Brachypodium* (XP_003558722) and rice (XP_015690912), respectively. This result was consistent with the findings in the stated phylogeny and classification of plants. All these proteins contain the conserved zf-Dof domain (Supplementary Figure S1).

### 2.2. Tissue-Specific Gene Expression

In order to analyze the expression of *PheDof12-1* in different tissues (root, stem, leaf, flowering leaf, flower) and floral organs (pistil, stamen, embryo, glume, palea, flower bud, bract), RNA was isolated to perform qRT-PCR. The results show that the transcription level of *PheDof12-1* in flowering leaf is significantly higher than in other tissues. In different flower organs, the expression of *PheDof12-1* was highest in palea, and lowest in bract (Figure 1C). In developing flowers, *PheDof12-1* had higher transcript accumulation at the floral bud formation stage (F1) (Figure 1D), and decreased gradually at flower development, which was consistent with the previously reported detection of *PheDof1* at early stages of flower formation and development [29]. We further generated *Pro_PheDof12-1_*-*GUS* transgenic lines, and glucuronidase (GUS) staining was detected in the vasculature of cotyledons and hypocotyls, true leaves, roots, flower, and pollen (Figure 1E,F). The results demonstrate that *PheDof12-1* is expressed in different tissues and at different flower development stages, suggesting that it is dynamic during plant development and may play an important role in moso bamboo growth and development.

### 2.3. Expression Patterns of PheDof12-1 under Stress Treatments

**Figure 2:**
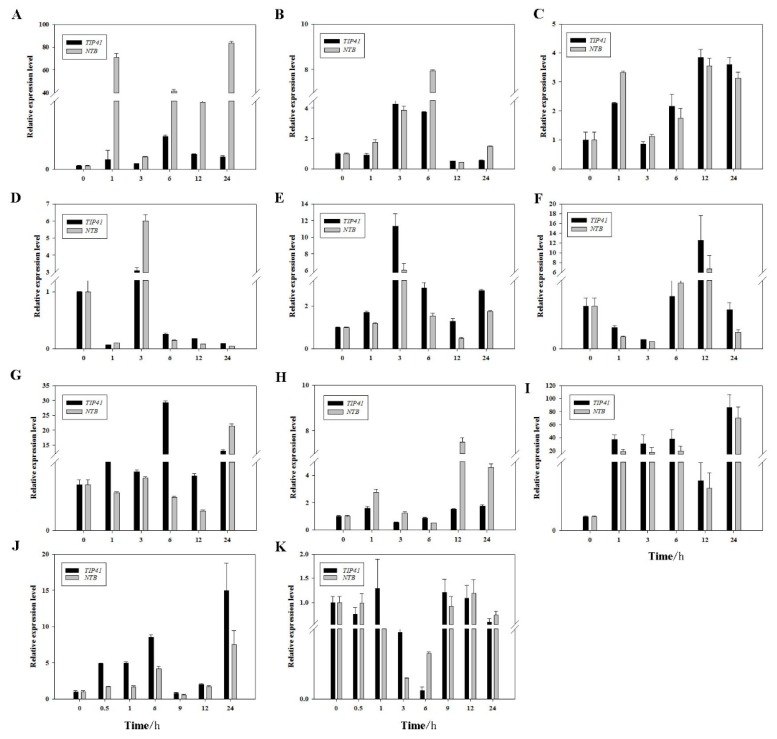
Relative expression of *PheDof12-1* in different tissues of moso bamboo under drought: (**A**) root, (**B**) stem, (**C**) leaf; salt: (**D**) root, (**E**) stem, (**F**) leaf; under cold: (**G**) root, (**H**) stem, (**I**) leaf; and under (**J**) gibberellin A3 (GA_3_) and (**K**) abscisic acid (ABA) treatments.

Previous reports have shown that Dof TFs are involved in abiotic stress [30]. To determine the expression pattern of *PheDof12-1* in moso bamboo under different stresses, we performed detailed qRT-PCR with *TIP41* and *NTB* as internal reference genes. The results show that *PheDof12-1* was responded to cold, drought, and salt stresses. In drought stress, *PheDof12-1* was induced and upregulated at each time point, and levels of transcripts in leaves and stems were slightly elevated, but a sharp increase occurred after 1 h in roots, peaking at 70.9-fold. This implies that *PheDof12-1* is induced and has a positive function in response to drought stress (Figure 2A–C). In cold treatment using *NTB* as a reference gene, the expression of *PheDof12-1* rapidly increased in leaves, reaching 86.1-fold at 24 h (Figure 2I). Regarding salt treatment, the maximum increase was observed at 12 h, reaching 12.5-fold in leaves when *TIP41* was used as the reference gene (Figure 2F), but the expression level was first induced and then decreased in roots. To further investigate the functions of *PheDof12-1*, we initially analyzed the effects of gibberellin A3 (GA_3_) and abscisic acid (ABA) on its expression (Figure 2J,K). In GA_3_ stress, the transcription level of *PheDof12-1* was induced and upregulated at almost every time point, peaking at 15.0-fold at 24 h. Under ABA treatment, the translation level of *PheDof12-1* initially decreased and then increased, was lowest at 6 h, dropping to undetectable levels, and reached a peak at 48 h. All of these data indicate that *PheDof12-1* takes part in the hormones and different abiotic stresses of moso bamboo.

### 2.4. Overexpression of PheDof12-1 Promotes Early Flowering in Arabidopsis

**Figure 3:**
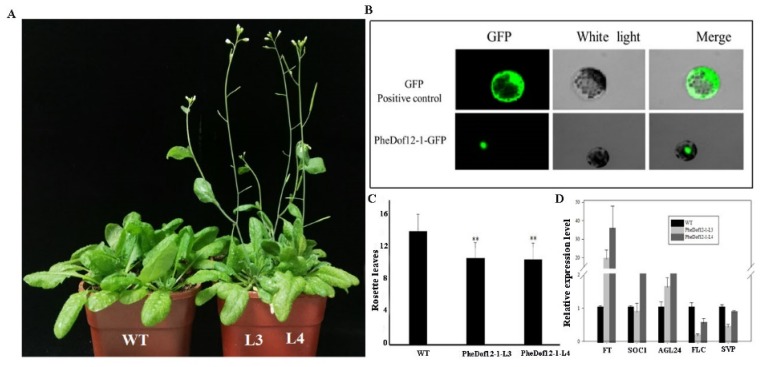
Analysis of an early flowering phenotype by overexpression of PheDof12-1 in Arabidopsis. (**A**) Phenotypes of overexpressing *PheDof12-1* transgenic lines (L3, L4) and wild-type (WT) plants as control under long-day (LD) conditions. (**B**) Subcellular localization of PheDof12-1. (**C**) Flowering time scored as number of rosette leaves at flowering of wild-type and transgenic plants under LD conditions. (**D**) Transcription levels of *FT*, *SOC1*, *AGL24*, *FLC*, and *SVP* in wild-type and transgenic plants. Arabidopsis *Actin* was used as the internal reference gene. Error bars indicate standard deviations. Asterisks indicate statistically significant difference between wild-type and transgenic plants (*p* < 0.01 by Student’s *t*-test).

In order to verify the subcellular localization of PheDof12-1, we further amplified its coding region and fused it to the N-terminal of the eGFP vector. The subcellular localization assay indicated that PheDof12-1 was localized in the nucleus, in accordance with its function as a transcription factor (Figure 3B). To study the genetic functions of *PheDof12-1*, we transformed it in Arabidopsis. The overexpressed plants showed an early flowering phenotype under LD conditions (Figure 3A), whereas *PheDof12-1* overexpression had no effect on flowering time under SD conditions (not shown). The flowering time was about 10 days earlier than wild-type, and the number of rosette leaves of overexpressed lines was smaller than that of wild Arabidopsis (Figure 3C). We further investigated the transcription levels of *FT*, *SUPPRESSOR OF OVEREXPRESSION OF CONSTANS1* (*SOC1)*, *AGAMOUS-LIKE 24* (*AGL24)*, *FLOWERING LOCUS C* (*FLC*), and *SHORT VEGETATIVE PHASE* (*SVP*) in the T3 generation to ascertain the downstream effects of this construct. *FT*, *SOC1*, and *AGL24* were upregulated, while *FLC* and *SVP* expression were rather low compared with wild-type (Figure 3D). These data suggest that *PheDof12-1* might regulate flowering by controlling the expression of *FT*, *SOC1*, *AGL24*, *FLC*, and *SVP*.

### 2.5. PheDof12-1 Interacts with Photoperiod-Related Regulators

**Figure 4:**
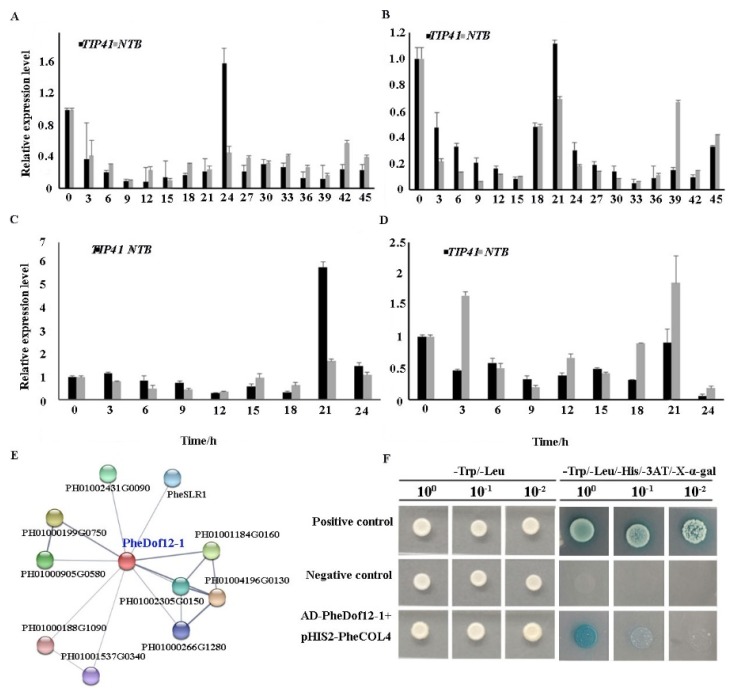
PheDof12-1 protein binds to the promoter region of *PheCOL4*. Relative expression of *PheDof12-1* under (**A**) LD and (**B**) SD conditions. Transcription level of *PheCOL4* under (**C**) LD and (**D**) SD conditions. (**E**) Interaction network of PheDof12-1 in moso bamboo. Colored balls (protein nodes) in the network were used as a visual aid to indicate different input proteins and predicted interactions. Enlarged protein nodes indicate the availability of 3D protein structure information. Gray lines connect proteins that are associated by recurring text mining evidence. (**F**) Yeast one-hybrid (Y1H) assay for AD-PheDof12-1 and pHIS2-PheCOL4. The reporter pHIS2 vector carrying the corresponding fragment and the effector AD-PheDof12-1 vector were cotransfected into yeast Y187 cells. Growth of the transfected yeast cells on a 3-AT and X-α-Gal medium indicates that the PheDof12-1 protein can bind to the PheCOL4 promoter.

In Arabidopsis, CDFs are transcriptional repressors that bind to *CO* and *FT* promoters to repress their transcription [20]. To explore whether PheDof12-1 can form heterodimers with other proteins, an interaction prediction was performed using STRING (https://stringdb.org/) based on the interaction network of rice orthologous genes. As shown in Figure 4E, PheDof12-1 interacted with 10 identified proteins. Among them, the B-box protein (PH01004196G0130), Dof transcription factor (PH01001184G0160), grain size gene (*PheSLR1*) [31], photoperiodic flowering response gene (*PH01002431G0090*) [32], and drought-induced protein (PH01000199G0750) were identified, suggesting that PheDof12-1 may be involved in growth and development, photoperiodic response, and abiotic stress.

*CDF1*, *CDF2*, *CDF3*, and *CDF5* had high mRNA levels at the beginning of the light period in Arabidopsis [21], and CDFs displayed a similar expression pattern in *Populus* [33]. So, we detected the expression patterns under photoperiod treatments. The results show that *PheDof12-1* was similarly expressed under both LD and SD conditions. The transcription level of *PheDof12-1* decreased with the increased light time, reaching the minimum value before dark (Figure 4A,B), with high mRNA levels at the beginning of the light period, which was consistent with the expression patterns of CDFs in Arabidopsis and *Populus*. The highly similar expression pattern of CDFs in *Populus*, Arabidopsis, and moso bamboo suggests a functional conservation.

CO and CO-like (COL) proteins are members of the B-box family, playing a central role in the photoperiod response pathway by mediating between the circadian clock and the floral integrators [34]. CDFs are transcriptional repressors that bind to the *CO* promoter to repress its transcription [20]. PheDof12-1 interacted with B-box proteins by interaction prediction; moreover, *PheDof12-1* and *PheCOL4* had similar expression patterns under photoperiod treatments (Figure 4C,D), suggesting that PheDof12-1 may interact with PheCOL4 in moso bamboo. To examine whether the PheDof12-1 protein regulated *PheCOL4* expression by directly binding to the promoter region, the *PheCOL4* promoter sequence was investigated. We performed a targeted yeast one-hybrid (Y1H) assay using PheDof12-1, and PheCOL4 was inserted upstream of the reporter plasmid pHIS2 and cotransfected into the yeast cells with the AD-PheCOL12-1 effector plasmid. The binding of PheCOL12-1 and the promoter of *PheCOL4* was indicated by the growth of transfected yeast cells on a nutrient-deficient medium (synthetic dextrose (SD)/-Trp-Leu-His) plus 3-amino-1, 2, 4-triazole (3-AT) and 5-bromo-4-chloro-3-indoxyl-α-D-galactopyranoside (X-α-Gal). The results show that all transformants tested were found to grow well on the SD/-Leu/-Trp medium when transferred onto SD/-Trp/-Leu/-His/3-AT/X-α-Gal plates for 3 days; only the yeast cells of AD-PheDof12-1 + pHIS2-PheCOL4 vectors and the positive control grew strong and turned blue (Figure 4F). This result suggests that PheDof12-1 could bind to the promoter of PheCOL4 and regulate *PheCOL4* expression in moso bamboo.

## 3. Discussion

Moso bamboo is a perennial plant characterized by rapid growth and a long vegetative stage that lasts for decades or even longer before flowering [25]. Dof proteins are a group of plant-specific TFs that are involved in diverse plant-specific biological processes [16]. In addition, recent research on *Dofs* is mainly in annual plants, and is limited in perennials. Therefore, researching the roles of *Dofs* in moso bamboo is necessary. In this study, a *Dof* gene, *PheDof12-1*, is identified from moso bamboo as a nucleus-localized transcription factor that contains typical zf-dof domains.

In recent decades, reports have indicated that Dof transcription factors are involved in stress response. In Arabidopsis, the expression level of *AtCDF3* is upregulated by cold, drought, high salinity, and ABA treatment [30], and overexpression of *35S::SlCDF1* and *35S::SlCDF3* increases Arabidopsis’s tolerance to salt and drought stresses [18]. In wheat, *TaDof14* and *TaDof15* are significantly induced under drought treatment [35]. Previous research has suggested that drought or other environmental stresses are functional in the flowering stage of bamboo, and the transcription levels of *Dof* genes are upregulated in drought stress [36]. In addition, studying the tolerance of *PheDof12-1* will help to characterize moso bamboo cultivars such as salt, cold, and drought tolerance. In this study, *PheDof12-1* exhibited differential expression patterns under the conditions of drought, cold, salt, and ABA and GA_3_ treatments. Through the drought, cold, salt, and GA_3_ stresses, the expression pattern of *PheDof12-1* is basically upregulated in roots, stems, and leaves, indicating that it might participate in abiotic stress and hormone pathways, which is consistent with previous reports [36,37]. The results provide a better understanding of the stress tolerance of *PheDof12-1* in moso bamboo.

*Hd1*/*CO* and *Hd3a*/*FT* are conserved genetic pathways that regulate photoperiodic flowering between rice and Arabidopsis by their genomic comparison [38]. In Arabidopsis, *CDF1*–*CDF3* are suggested to participate in photoperiodic flowering [39]. *JcDof3* is a circadian clock regulated gene involved in the regulation of flowering time in *Jatropha curcas* [40]. In rice, *OsDof12* and *CDF1* belong to the same group [41], and overexpression of *OsDof12* resulted in early flowering by increasing the expression of *Hd3a* and *OsMADS14* under LD conditions [22]. *PheDof12-1* is the homologous gene of *OsDof12*, and Dof-Hd3a-MADS-flowering may play an important role in moso bamboo flowering [36]. Therefore, we researched the function of *PheDof12-1* in flowering time by ectopic expression in Arabidopsis for the first time, and the transgenic lines overexpressing *PheDof12-1* show earlier flowering than the wild-type plants under LD conditions. In addition, *FT*, *SOC1*, and *AGL24* are upregulated and *FLC* and *SVP* are downregulated in the transgenic lines. *FT* promotes flowering [42], which is activated by *CO* in the phloem [43]. SOC1 is a core regulator of flowering in Arabidopsis, which can interact with SVP and AGL24 proteins, but SVP and AGL24 have opposite effects on flowering time, acting as floral repressor and inducer, respectively [44]. *FLC* encodes a MADS domain-containing transcription factor that acts as an inhibitor of flowering [45]. This leads us to suspect that *PheDof12-1* promotes flowering time by regulating *FT*, *SOC1*, *AGL24*, *FLC*, and *SVP* directly or indirectly, suggesting that it might retain some function in the control of flowering time through similar molecular mechanisms to those observed when expressed in Arabidopsis.

Diurnal oscillation of the transcription levels of *CDFs* has been reported in Arabidopsis and other species [21,23]. In Arabidopsis, *CDF1*–*CDF3* and *CDF5* show maximum expression at the beginning of the light period, decreasing to a minimum between 16 and 20 h, then rising again during dawn [21]. In tomato, *SlCDF1* and *SlCDF3* exhibit maximum expression at the beginning of the day, while *SlCDF2*, *SlCDF4*, and *SlCDF5* exhibit maximum levels during the night [18]. In rice, *OsDof12* is strongly inhibited by dark treatment [22]. In the study, *PheDof12-1* exhibited significantly diurnal expression patterns with high mRNA levels at the beginning of the light period under LD and SD conditions, supporting the assumption that it is a true homologue of the Arabidopsis CDFs. In Arabidopsis, CDFs can bind to the *CO* promoter to repress its transcription [20], and PttCDF3 can bind directly to the *PttCO2* promoter in *Populus* [33]. In moso bamboo, the diurnal expression pattern of *PheCOL4* is consistent with *PheDof12-1*, and Y1H analysis shows that PheDof12-1 binds directly to the promoter of *PheCOL4*. These results support the hypothesis that flowering regulator CO, a target of CDFs, is controlled precisely [21], which is similar to the situation in Arabidopsis and *Populus*.

## 4. Materials and Methods

### 4.1. Plant Materials and Treatments

Moso bamboo seeds were harvested from Guilin in the Guangxi Zhuang Autonomous Region, China. Seedlings were grown in an illumination incubator under long-day conditions (16 h light/8 h dark) at day/night temperatures of 25/18 °C, and watered with Hoagland nutrient solution. For drought and salt stress, the seedlings were watered with 50% Hoagland’s solution with 20% polyethylene glycol 6000 (PEG 6000) and 250 mM NaCl. For low temperature treatment, the plants were transferred to a growth chamber at 4 °C, and plant leaf, stem, and root tissues were collected [46]. For abscisic acid (ABA) and gibberellin A3 (GA_3_) treatments, the seedlings were watered with 200 µM ABA [47] and 200 µM GA_3_ solution [48]. To detect the transcriptional level of *PheDof12-1* in photoperiod treatments, leaves were collected for analysis from plants exposed to LD (16 h light/8 h dark) and SD (16 h light/8 h dark) treatments [21]. All samples were immediately frozen in liquid nitrogen and stored at −80 °C until further analysis.

### 4.2. Bioinformatic Analysis

The sequences were downloaded from BambooGDB (http://forestry.fafu.edu.cn/db/PhePacBio/) [49]. Molecular weight (MW) and isoelectric point (pI) were analyzed using ProtParam (http://web.expasy.org/protparam/) [50]. The structure was shown using Gene Structure Display Server software (http://gsds1.cbi.pku.edu.cn/index.php) [51]. To search the database, the Basic Local Alignment Search Tool (BLAST) network service from the National Center for Biotechnology Information (NCBI) web server was applied. Homologue alignment was obtained using Clustal 1.83, and a phylogenetic tree was constructed by MEGA6.0 [52] using the following parameters: NJ method, complete deletion, and bootstrap with 1000 replicates.

### 4.3. Vector Construction and Plant Transformation

The subcellular localization was performed by transfecting GFP-tagged PheDof12-1 into Arabidopsis sheath protoplasts [53] (Supplementary Table S1). The full-length cDNA of *PheDof12-1* was fused in frame with the GFP cDNA and ligated between the CaMV 35 S promoter and the nopaline synthase terminator. The fluorescence signals were examined using a confocal laser scanning microscope (Leica Microsystems, Wiesler, Germany).

The full-length coding sequence of *PheDof12-1* was cloned into the pCAMBIA 2300 vector under the control of the modified CaMV 35S promoter (Supplementary Table S1). The pCAMBIA 2300-*PheDof12-1* vector was introduced into *Agrobacterium umefaciens* strain GV3101 for Arabidopsis transformation in the Col-0 background by the floral dipping method [54]. Putative transgenic plants were screened on 50% Murashige and Skoog (MS) solid medium supplemented with 50 mg/L kanamycin, and homozygous T3 or T4 seeds were used.

In order to analyze the spatial expression patterns of PheDof12-1, a 2 kb region upstream of the PheDof12-1 transcription start site was cloned and fused to the pCAMBIA2391Z vector to generate the *Pro_PheDof12-1_*-*GUS* reporter, which was transformed into wild-type (WT) plants (Supplementary Table S1). For GUS staining, *Pro_PheDof12-1_*-*GUS* transgenic plants were used as previously reported [55].

### 4.4. Gene Expression Analysis

Total RNA was extracted from the frozen samples using Trizol reagent (Invitrogen, Carlsbad, CA, USA) according to the manufacturer′s instructions and treated with DNase I (TaKaRa, Tokyo, Japan) to remove genomic DNA contamination. Then, for each sample, the first-strand cDNA was synthesized using a PrimeScript™ RT Reagent Kit (TaKaRa). The expression profiles of PheDof12-1 in different tissues, abiotic stress, and photoperiod treatments were analyzed by quantitative RT-PCR (qRT-PCR). *TIP41* and *NTB* were used as internal housekeeping genes [56]. The qRT-PCR reactions were carried out using a Light Cycler 480 System (Roche, Basel, Switzerland) and a SYBR Premix EX TaqTMkit (Roche, Mannheim, Germany). All reactions were performed in triplicate, both technical and biological, and data were analyzed using the Roche manager software. The primer sequences are listed in Supplementary Table S1.

### 4.5. Yeast One-Hybrid Assay

To perform the Y1H assay, the full length of PheDof12-1 was cloned into the pGADT7-Rec2 bait vector, and the promoter sequence of PheCOL4 was cloned into the pHIS2 prey vector (Supplementary Table S1). The lithium acetate method was used to transform into the Y187 strain. The transformed yeast cells were selected on SD/-Trp/-Leu and SD/-Trp/-Leu/-His/-3AT/X-α-Gal plates at 30 °C for 3–5 days.

## 5. Conclusions

In conclusion, the present study provides new notions about the function of Dof TFs in moso bamboo and shows PheCOL12-1 as a key factor with multiple roles related to abiotic stress, and the developmental program underlying the transition from the vegetative to the reproductive phase under LD conditions. PheCOL12-1 is a nucleus-localized transcription factor that regulates photoperiodic-related regulators. These findings not only increase our understanding of the functional roles of Dof proteins in the regulation of abiotic stress and flowering time, but also provide an important candidate gene for studying molecular regulation mechanisms of moso bamboo flowering.

## Supplementary Materials

Supplementary materials can be found at http://www.mdpi.com/1422-0067/20/2/424/s1.

## Abbreviations

LDs	Long days
SDs	Short days
DOF	DNA binding with One Finger
PCR	Polymerase chain reaction
CO	CONSTANS
FT	Flowering locus T
TFs	Transcription Factors
GI	GIGANTEA
FKF1	FLAVIN-BINDING, KELCH REPEAT, F-BOX1
CDF	Cycling Dof Factor
Hd3a	Heading date 3a
MADS	MCM1, AGAMOUS, DEFICIENS and SRF
GFP	Green Xuorescent protein
ABA	Abscisic acid
GA	Gibberellin
GUS	Glucuronidase
COL	CO-Like
FLC	FLOWERING LOCUS C
SOC1	SUPPRESSOR OF OVEREXPRESSION OF CONSTANS1
SVP	SHORT VEGETATIVE PHASE
AGL24	AGAMOUS-LIKE 24
3-AT	3-amino-1, 2, 4-triazole
X-α-Gal	5-bromo-4-chloro-3-indoxyl-α-D-galactopyranoside
