# Supplementary material for: Multifaceted Role of PheDof12-1 in the Regulation of Flowering Time and Abiotic Stress Responses in Moso Bamboo (Phyllostachys edulis)
Source: Int J Mol Sci. 2019 Jan 19;20(2):424. doi: 10.3390/ijms20020424 (PMC6358834; doi:10.3390/ijms20020424)
Supplement: Supplementary file 1 [file ijms-20-00424-s001.pdf]

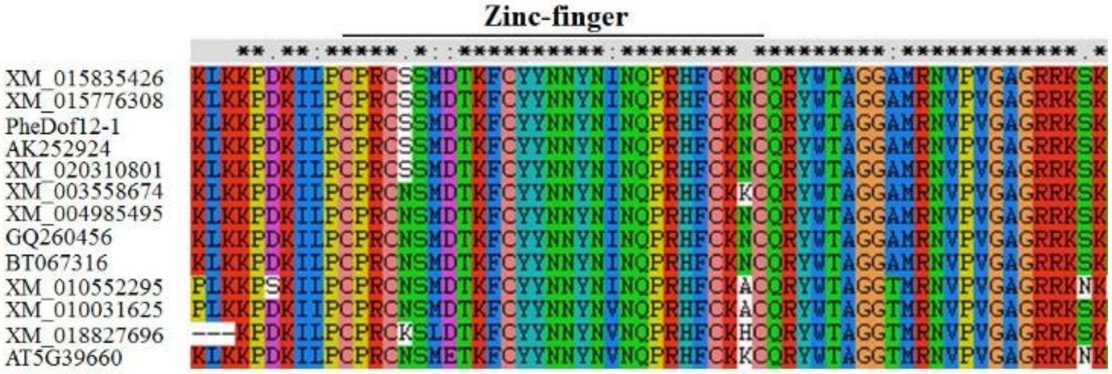

Figure. S1. Multiple alignment of deduced amino acid sequences of Dof proteins among different plants. The putative signal peptide and conservative C2C2-type zinc-finger motif at N-terminal end are labeled. The highly conserve C residues in N-terminal are shaded in pink.

Table 1**.** primers were used for gene cloning and qRT-PCR.

Gene		Primer sequence (5′→3′)
2300-PheDof12-1	Forward	GGAATTCATGGGGGCGTGCAGGGCAG
	Reverse	GGGTACCTCAAGATCCCTCTTGGAAGGTCACTG
GFP-PheDof12-1	Forward	GGGTACCATGGGGGCGTGCAGGGCAG
	Reverse	GCGTCGACAGATCCCTCTTGGAAGGTCACTG
AD-PheDof12-1	Forward	GCAGAGTGGCCATTATGGCCCATGGGGGCGTGCAGGGCAG
	Reverse	GCGGCCGACATGTTTTTTCCCTCAAGATCCCTCTTGGAAGGTCACTG
qRT-PheDof12-1	Forward	AGGGCAGCGGCAGGAGGA
	Reverse	TTTCTTGTACTTTTGGTTCCGTCGT
pHIIS-PheCOL4	Forward	CGAATTCCCGGGGAGCTCAACTGCTCTTCCGATTAAATGTCTAC
	Reverse	CGCGGATCGATTCGCGAAGTCTAGACTTCTAATGCAAGCGTTT
qRT-PheCOL4	Forward	ACTCCATGAACCACAGCGTA
	Reverse	GAATGGTCTTCTCGAACCGC
TIP41	Forward	AAAATCATTGTAGGCCATTGTCG
	Reverse	ACTAAATTAAGCCAGCGGGAGTG
NTB	Forward	TCTTGTTTGACACCGAAGAGGAG
	Reverse	AATAGCTGTCCCTGGAGGAGTTT
Actin	Forwards	GTATGTGGCTATTCAGGCTGT
	Reverse	CTGGCGGTGCTTCTTCTCTG
FT	Forward	CTTGGCAGGCAAACAGTGTATGCAC
	Reverse	GCCACTCTCCCTCTGACAATTGTAGA
SOC1	Forward	AGCTGCAGAAAACGAGAAGCTCTCTG
	Reverse	GGGCTACTCTCTTCATCACCTCTTCC
AGL24	Forward	GAGGCTTTGGAGACAGAGTCGGTGA
	Reverse	AGATGGAAGCCCAAGCTTCAGGGAA
FLC	Forward	AGCCAAGAAGACCGAACTCA
	Reverse	TTTGTCCAGCAGGTGACATC
SVP	Forward	CAAGGACTTGACATTGAAGAGCTTCA
	Reverse	CTGATCTCACTCATAATCTTGTCAC
